# Editorial: Microalgae Biology and Biotechnology

**DOI:** 10.3389/fpls.2020.628267

**Published:** 2021-01-18

**Authors:** Dimitris Petroutsos, Lutz Wobbe, EonSeon Jin, Matteo Ballottari

**Affiliations:** ^1^Univ. Grenoble Alpes, CNRS, CEA, INRAe, IRIG-LPCV, Grenoble, France; ^2^Center for Biotechnology (CeBiTec), Faculty of Biology, Bielefeld University, Bielefeld, Germany; ^3^Department of Life Science, Hanyang University, Seoul, South Korea; ^4^Department of Biotechnology, University of Verona, Verona, Italy

**Keywords:** microalgae, cyanobacteria, genetic engineering, terpenoids, triacylglycerols, CRISPR-Cas9

The interest for industrial exploitation of microalgae dates back to the end of the last century. The combination of the light energy-capturing ability of photosynthesis with the high yields of controlled microbial cultivation make microalgae potentially valuable organisms for economical, industrial-scale production processes in areas including nutrition, aquaculture, pharmaceuticals, and biofuels. Moreover, microalgae allow to produce biomass even in not arable land, exploiting the light energy available marginal areas. Several national and international research programs were deeply involved in the investigation of microalgae biodiversity and their characterization. More recently, strong efforts were reported by the scientific community for the development of biotechnological tools and omics data required to domesticate and improve microalgae strains for their applicative use. Several microalgal species were indeed included among the possible “novel food” sources for human consumption and some strains are nowadays considered for high-value metabolites to be used in the cosmetic sector, for fish/animal feed, for the production of bioplastics, drugs, or other commodities. Microalgae also have a high potential for environmental applications, being used as biostimulants, fertilizers and/or biopesticides in agriculture, reducing the negative environmental impact of fertilizers and pesticides. Finally, the potential use of microalgae biomass for biofuels has been considered, even if more research efforts are still required to achieve sustainability of this application.

This Research Topic highlights recent advances in the comprehension of microalgae biology, which is necessary to design strategies for their biotechnological application. Despite the great biodiversity of microalgae, most of the research described is focused on a few tens' species. The work by Wang et al. proved an example of light-driven changes of phytoplankton population in an eutrophic highland lake in China. Significant positive relationships were observed between taxonomic diversity and functional diversity of phytoplankton, which were positively correlated with light conditions. Bioprospecting of natural species is an important task for the microalgae research communities in order to find new species and new metabolic features to expand the potential of microalgae for their biotechnological application. Kareya et al. investigated the molecular mechanisms of the CO_2_-driven carbon partitioning in the marine oleaginous microalgae *Microchloropsis gaditana* NIES 2587. The results obtained identified the key metabolites and the key metabolic pathways affected by high/low availability of CO_2_: this information could then be used to re-direct the carbon flow to improve the metabolic efficiency of target metabolic products. The influence of the environmental conditions on the cell functions was also investigated in detail by Kuo et al., where the role of nitric oxide in the regulation of autophagy in *Chlamydomonas reinhardtii* was investigated. Autophagy and cell death are important events occurring in stress conditions that significantly influence biomass and metabolite productivity of microalgal cultures: the work herein reported demonstrates that nitric oxide mediated autophagy under lethal high light intensity. Resistance to high light and photooxidative stress is mainly related to antioxidants accumulation. In the work herein reported by Kim et al., the resistance to ROS was improved in the cyanobacterium *Synechococcus elongatus* PCC 7942 by overexpression of the enzyme Dehydroascorbate reductase (DHAR) which catalyzes the glutathione-dependent reduction of oxidized ascorbate. Carotenoids are other antioxidants strongly involved in the mitigation of oxidative stress. Carotenoids are produced in microalgal cells by the terpenoid biosynthetic pathway. Feng et al. report the crystal structure at 2.7Å of the cyanobacterial Geranylgeranyl Pyrophosphate Synthase (CrtE), one of the key enzymes involved in the formation of the terpene precursors, identifying also some specific residues involved in the catalytic activity.

Genetic engineering of microalgal strains requires specific and advanced tools, which are still lacking in many cases. Among the most critical tools for a successful modification of microalgal genomes are efficient and reliable selection markers, to be used for screening of transformed/mutated/edited colonies. In the study conducted by de Carpentier et al., a novel marker for *Chlamydomonas reinhardtii* is developed based on the resistance to the antibiotic blasticidin S, using the *Bacillus cereus* blasticidin S deaminase (BSR) gene. The identification of new selectable markers for the modification of microalgal genomes is important considering the endogenous resistance to specific antibiotics observed in several species, and the possibility to combine different constructs in a single strain, requiring multiple selection markers. Synthetic biology application requires, indeed, insertion of usually multiple genes in microalgae. As described in Shahar et al., the expression of heterologous genes in *C. reinhardtii* is strongly dependent on the copy number of the gene of interest. The possibility to generate site-specific mutants and gene overexpression in *C. reinhardtii* is here described by Kim et al., where an advanced method based on CRISPR-CAS9 mediated non-homologous end joining (NHEJ) is reported. The method reported is based on recombinant ribonucleoprotein CAS9 assembled *in vitro* and electroporated in *C. reinhardtii* cells potentially allow to generate mutants in any non-lethal gene. Moreover, it can be used to express a heterologous gene of interest avoiding position-effects. Besides important milestones being reached for the genetic engineering of several microalgal strains, in many cases transformation protocols still need to be set-up and optimized. In the case of the cyanobacterium *Synechocystis* multiple genome copies are present per cell, with the consequence that segregating mutations across all genome copies can be time-consuming: Pope et al. are here presenting a novel transformation protocol in the case of *Synechocystis* sp. PCC 6803 involving phosphate depletion which strongly reduce the time required to obtain fully segregated mutants.

Finally, this Research Topic reports some recent biotechnological application of specific microalgae strains for the production of target metabolites. The work by Hammel et al. describes a successful approach to improve biomass productivity in *C. reinhardtii* by overexpression of one of the Calvin-Benson cycle enzyme, the sedoheptulose-1,7-bisphosphatase (SBP1) which catalyzes a rate- limiting step of this carbon fixation cycle. Overexpression of SBP1 increases biomass yield and photosynthetic efficiency, thus representing a promising genetic engineering strategy for other microalgal species. Improvement of carbon fixation reactions requires also an efficient carbon storage capacity in order to be effective. In the work herein reported by Li et al. a strong increase in starch and biomass content was achieved in *C. reinhardtii* by overexpression of the regulatory gene Gcn5-Related N-Acetyltransferase, paving the way for future application of this regulatory system to boost starch accumulation. One of the other main macromolecules found in microalgal cells with storage function is lipids. The interest for lipids produced in microalgae is extremely high due to their potential use as biofuels or food feedstocks. In the work published by Muñoz et al. three enzymes involved in the Kennedy pathway, namely glycerol-3-phosphate acyltransferase (GPAT), lysophosphatidic acid acyltransferase (LPAT), and diacylglycerol acyltransferase (DGAT), which catalyze key steps in the formation of triacylglycerol, were overexpressed in *Neochloris oleoabundans*. The Kennedy pathway is the key metabolic process, required for the accumulation of triacylglyceroles (TAGs) in microalgal cells. The phenotype of single GPAT, LPAT, or DGAT overexpressor was characterized by an increased cellular TAG content without having a significant impact on the growth rate of the strains. Finally, in this Research Topic, genetic engineering approaches targeting cyanobacteria are reported showing the feasibility of D-lactate production by editing the photosynthetic alternative electron transport flows in a strain expressing a variant of the *C. reinhardtii* D-lactate dehydrogenase and an *E.coli* lactate permease (Selão et al.). Cyanobacteria are also here reported by Betterle et al., as an efficient platform for the production of biopharmaceutical and biotherapeutic proteins, demonstrating the possibility to produce interferon alpha-2 (IFN) protein up to 12% of total cellular protein in soluble form. IFN is a member of the Type I interferon cytokine family, well-known for its antiviral and anti-proliferative functions: the results achieved by Betterle et al. pave the way for sustainable production of biopharmaceutical proteins, including those with antiviral properties, by microalgae cultivation. Another class of important biomolecules.

In conclusion, this Research Topic covers representative aspects of the ongoing research on microalgae biology and biotechnology; these aspects span from the investigation of the molecular mechanisms of key cell functions to the development of advanced tools for genetic engineering, which collectively may allow to produce high-value products as food supplements, biopharmaceutical and biotherapeutic proteins or potentially biofuels.

## Author Contributions

MB draft the editorial text. DP, LW, and EJ revised and approved the final version of the editorial text. All authors contributed to the article and approved the submitted version.

## Conflict of Interest

The authors declare that the research was conducted in the absence of any commercial or financial relationships that could be construed as a potential conflict of interest.

